# RNA-binding protein MBNL2 mitigates neuropathic pain after chemotherapy through destabilizing CCR2 expression in primary sensory neurons

**DOI:** 10.1016/j.neurot.2026.e00905

**Published:** 2026-04-11

**Authors:** Keshi Yan, Ruining Ma, Bing Wang, Xiaozhou Feng, Xinju Li, Xianglei Meng, Huijie Shang, Benjamin M. Kidd, Maurice S. Swanson, Huijuan Hu, Yuan-Xiang Tao

**Affiliations:** a Department of Anesthesiology, New Jersey Medical School, Rutgers, The State University of New Jersey, Newark, NJ 07103, USA; b Department of Molecular Genetics and Microbiology, Center for NeuroGenetics and the Genetics Institute, University of Florida College of Medicine, Gainesville, FL 32610, USA; c Department of Physiology, Pharmacology & Neuroscience, New Jersey Medical School, Rutgers, The State University of New Jersey, Newark, NJ 07103, USA; d Department of Cell Biology & Molecular Medicine, New Jersey Medical School, Rutgers, The State University of New Jersey, Newark, NJ 07103, USA

**Keywords:** Muscleblind-like protein 2, C–C chemokine receptor type 2, Paclitaxel, Dorsal root ganglion, And chemotherapy-induced neuropathic pain

## Abstract

Chemotherapy drug-induced changes of gene expression in the dorsal root ganglion (DRG) are critical for the genesis of chemotherapy-induced neuropathic pain (CINP). However, the mechanisms driving these changes remain elusive. Here, we report the downregulation of muscleblind-like protein 2 (MBNL2), an RNA-binding protein, in the DRG neurons after intraperitoneal injection of paclitaxel. Rescuing this downregulation blocks an increase of the C–C chemokine receptor type 2 (CCR2) in the DRG and mitigates paclitaxel-induced mechanical allodynia, heat and cold hyperalgesia and ongoing pain. Conversely, DRG downregulation of MBNL2 increases the expression of CCR2 in the DRG neurons and leads to CINP-like symptoms in naïve mice. Mechanistically, paclitaxel-induced downregulation of MBNL2 reduces its binding to the 3′-untranslated region of *Ccr2* mRNA, thereby enhancing the stability of *Ccr2* mRNA in the DRG. Given that MBNL2 and CCR2 are co-expressed in DRG neurons, these findings suggest that MBNL2 alleviates CINP, likely by destabilizing CCR2 expression in the DRG, and may represent a promising therapeutic strategy for this condition.

## Introduction

Chemotherapy drugs cause nerve damage, leading to chemotherapy-induced neuropathic pain (CINP), one of the common adverse effects during cancer treatments [1]. It is characterized by pain, tingling, numbness, and other sensory and motor dysfunctions, often presenting symmetrically in a socking-glove distribution [2]. The incidence and severity of CINP correlate with the duration and dose of chemotherapeutic agents. Many patients ultimately discontinue treatment because of CINP, thereby adversely affecting survival [3]. Current management of CINP remains limited in part due to unclear mechanisms underlying this disorder. Chemotherapy drugs unrestrictedly access the dorsal root ganglion (DRG) neurons [4,5] and induce ectopic spontaneous activity in these neurons, which is considered to trigger CINP [[6], [7], [8], [9]]. This abnormal activity is associated with chemotherapy drug-induced changes in pain-related genes in the DRG [[6], [7], [8], [9]]. There is a pivotal need to exploit how chemotherapy drugs drive these molecular changes.

The chemokine (C–C motif) receptor 2 (CCR2) and its binding ligand, C–C motif chemokine ligand 2 (CCL2), contribute to the development and maintenance of neuropathic pain. CCL2 and CCR2 are upregulated in injured DRG neurons following peripheral nerve injury [[10], [11], [12]] or after systemic injection of chemotherapy drugs [13,14]. CCL2 directly excites injured small-, medium-, and large DRG neurons through CCR2 activation by autocrine/paracrine processes [[15], [16], [17], [18]]. CCR2 sensitizes nociceptors via transactivation of TRPV1 and Nav1.8 [15,[19], [20], [21]]. Genetic CCR2 knockout and intrathecal injection of the CCL2-neutralizing antibody, CCR2 antagonists, or CCR2 antisense oligodeoxynucleotides mitigated nociceptive hypersensitivity after peripheral nerve injury [15,[22], [23], [24], [25]] or following systemic injection of chemotherapy drugs [13,14,26]. Intrathecal administration of CCL2 led to mechanical allodynia and heat hyperalgesia [23,25]. These findings demonstrate that CCL2/CCR2-mediated signaling is critical for the genesis of neuropathic pain, including CINP. However, the mechanisms responsible for CCR2 upregulation in DRG neurons after chemotherapy remain unclear. Defining these mechanisms may reveal new therapeutic strategies for CINP.

Muscleblind-like (MBNL) proteins, a family of conserved multifunctional RNA-binding proteins, are key regulators of gene expression and RNA splicing [27]. The MBNL family consists of three closely related paralogs in mammals., MBNL1-3. MBNL1 and MBNL2 are widely expressed, whereas MBNL3 is detected mainly in the placenta [28]. MBNL1-3 are present in both the nuclei and cytoplasm of cells [29,30]. They bind to pre-mRNA in the nucleus to regulate alternative splicing [30], while in the cytoplasm they bind to 3′-untranslated region (3′-UTR) of mRNA to regulate mRNA expression [[30], [31], [32]]. Dysregulation of MBNL proteins has been linked to a variety of human diseases [33,34]. However, the function of MBNL proteins, particularly in the DRG and in the context of pain, remains unknown.

Here, we show that the intraperitoneal (i.p.) injection of the chemotherapy drug paclitaxel time-dependently downregulates the expression of MBNL2 protein in DRG neurons. This downregulation is responsible for the maintenance of paclitaxel-induced CINP by enhancing the stabilization of CCR2 expression in DRG neurons. Our findings identify MBNL2 as a potential analgesic regulator and therapeutic target in CINP.

## Methods

### Animal preparation

*Mbnl2*^f/f^ mice were provided by Dr. Maurice S Swanson (Department of Molecular Genetics and Microbiology, Center for NeuroGenetics and the Genetics Institute, University of Florida College of Medicine). Rosa26^*Mbnl2*^ conditional knock-in mice were generated at the Genome Editing Shared Resource, Rutgers-Cancer Institute of New Jersey. Advillin^Cre−ERT2/+^ mice were purchased from the Jackson Laboratory (Stock No: 026516). To generate sensory neuron-specific inducible conditional MBNL2 knockdown (cKD) mice, female *Mbnl2*^f/f^ mice were crossed with male advillin^Cre−ERT2/+^ mice. To generate sensory neuron-specific inducible conditional MBNL2 knock-in (cKI) mice, female Rosa26^*Mbnl2*^ mice were crossed with male advillin^Cre−ERT2/+^ mice. All adult male and female mice (2–3 months old, 25–30 g) were housed under standard conditions in the central housing facility at Rutgers New Jersey Medical School, maintained on a 12-h light/dark cycle with food and water provided ad libitum. To minimize inter- and intra-individual variability in behavioral outcomes, all animals were acclimated to the testing environment for 1–2 days before behavioral tests. Experimental procedures were approved by the Institutional Animal Care and Use Committee at Rutgers New Jersey Medical School and adhered to ethical guidelines from the US National Institutes of Health and the International Association for the Study of Pain. Mice were randomly assigned to different experimental groups, and experimenters were blinded to the treatment conditions. All efforts were made to minimize mouse suffering and reduce the number of animals used.

### Paclitaxel-induced CINP mouse model

The mouse model of paclitaxel-induced chemotherapy neuropathic pain (CINP) was established as previously described [[35], [36], [37], [38], [39]]. Briefly, paclitaxel (6 mg/ml in 50% Cremophor EL (Sigma-Aldrich, St. Louis, MO) and 50% ethanol (Sigma-Aldrich)) was diluted in sterile saline to a final concentration of 0.4 mg/ml. Mice were administered intraperitoneally with paclitaxel at a dose of 4 mg/kg every other day for a total of four injections on days 0, 2, 4, and 6, resulting in an 8-day treatment period. The vehicle was prepared identically without paclitaxel.

### DRG microinjection

DRG microinjection was conducted as described with minor modifications [[35], [36], [37], [38], [39]]. Before surgery, bupivacaine was injected at the incision site for local infiltration anesthesia. After the mouse was anesthetized with isoflurane, a dorsal midline incision was made in the lower lumbar region. The unilateral L3 and L4 articular processes were exposed and then removed. Viral solution (1 μl/DRG; 4–9 × 10^12^ VG/ml; dissolved in PBS) was injected into the unilateral L3/4 DRGs using a glass micropipette connected to a Hamilton syringe. After injection, the micropipette was retained in place for 10 min to ensure efficient delivery. The surgical field was irrigated with sterile saline, and the skin incision was closed with wound clips. None of the microinjected mice exhibited signs of paresis or other abnormalities.

### Behavioral testing

Behavioral testing, including mechanical, heat, and cold tests, was carried out sequentially with 1-h intervals as previously described [[35], [36], [37], [38], [39]]. Conditioned place preference (CPP) testing was performed at 2 or 3 weeks post-paclitaxel/vehicle or 3 weeks after tamoxifen injection, and locomotor functional testing was conducted before tissue collection.

Paw withdrawal frequencies (PWFs) in response to mechanical stimulation were measured using calibrated von Frey filaments (0.07 g and 0.4 g; Stoelting Co., Wood Dale, IL, USA). Mice were placed in Plexiglas chambers on an elevated mesh screen. Each filament was applied to the plantar surface of both hind paws for 10 times, with each trial lasting ∼1 s and repeated at 5-min intervals. Quick paw withdrawal with/without flinching/shaking or biting was considered as positive response, and the percentage of paw withdrawal frequency was calculated as the number of positive responses divided by 10 trials.

Paw withdrawal latency (PWL) in response to heat stimulation was evaluated using a Model 336 Analgesic Meter (IITC Inc./Life Science Instruments, Woodland Hills, CA, USA). Mice were placed in Plexiglas chambers on a glass plate, and radiant heat was applied to the mid-plantar surface of each hind paw. The latency, defined as the time from heat application to paw withdrawal, was recorded. Trials were conducted three times at 5-min intervals per side, with a 20-s cutoff to avoid tissue damage.

Paw withdrawal latency in response to cold stimulation was measured by placing mice in Plexiglas chambers on a cold aluminum plate maintained at 0 °C. The latency was defined as the time from placement on the plate to a nociceptive response, such as jumping or paw flinching. Each trial was repeated three times at 10-min intervals, with a 20-s cutoff to prevent tissue damage.

The CPP test was conducted using an apparatus with two Plexiglas chambers connected by an internal door (Med Associates Inc., St. Albans, VT, USA). Each chamber had distinct floor textures and wall patterns (e.g., rough floor with horizontal stripes vs. smooth floor with vertical stripes). Mice were preconditioned for 30 min with free access to both chambers to habituate to the environment. Baseline duration in each chamber was recorded over 15 min, and animals with significant pre-existing biases (>80% or < 20% of total time in one chamber) were excluded. During conditioning, mice received intrathecal injections of saline (5 μl) paired with one chamber in the morning, and lidocaine (0.8% in 5 μl saline) paired with the other chamber in the afternoon. This schedule was repeated for 3 days, alternating the order of saline and lidocaine injections each day. On the test day (≥20 h after the final conditioning), mice were placed in one chamber with free access to both. The time spent in each chamber was recorded over 15 min, and CPP scores were calculated as the difference between the test time and the preconditional time in the lidocaine-paired chamber.

Locomotor function was tested using three reflexes: placing, grasping, and righting reflexes. Briefly, placing reflex was assessed by placing the dorsal surfaces of the hind paws into contact with the edge of a table and recording whether the hind paws reflexively placed on the table surface. The grasping reflex was assessed by placing the animal on a wire grid and recording whether the hind paws grasped the wire upon contact. The righting reflex was evaluated by placing the animal on its back on a flat surface and recording whether it promptly returned to a normal upright position. Each trial/reflex was repeated 5 times at 5-min intervals, and the scores for each reflex were recorded based on counts of each normal reflex.

### Plasmid construction and virus production

The full-length sequence of *Mbnl2* cDNA was synthesized and amplified from total RNA of mouse DRGs using the SuperScript III One-Step RT-PCR System with the Platinum Taq High Fidelity Kit (Thermo Fisher Scientific) and gene-specific primers (Supplementary Table 1). The amplified PCR products were ligated into the *Bgl*II and *Bsr*BI restriction sites of the pAAV-CMV-MS vector (Cell Biolabs), replacing the enhanced GFP sequence. The control plasmid harboring enhanced green fluorescent protein (GFP) was prepared in parallel. A mouse *Mbnl2* shRNA duplex targeting nucleotides 984–1003 within the open reading frame of mouse *Mbnl2* mRNA (GenBank accession no. ENSMUST00000032745) was designed (Supplementary Table 1). A mismatch shRNA containing a scrambled sequence with no known homology to any mouse gene was used as a control (scrambled shRNA; Supplementary Table 1). The shRNA oligonucleotides were annealed and cloned into the pAAV-U6-shRNA vector (Cell Biolabs). DNA sequencing was performed to verify the integrity of the recombinant plasmids to ensure correct insertion and orientation.

To prepare AAV5 viral particles, the recombinant vectors described above plus the necessary packaging plasmids, including pHelper, were co-transfected into HEK293T cells (Takara, Mountain View, CA) using the PEI transfection method. 72 h later, the transfected cells were harvested, and viral particles were purified using the AAVpro Purification Kit (Takara, Mountain View, CA). The viral titer was determined using the AAV Real-Time PCR Titration Kit (Takara) according to the manufacturer's protocol. The resulting AAV5 vectors expressed mRNA under the control of the cytomegalovirus (CMV) promoter, while shRNA expression was driven by the U6 promoter.

### Quantitative real-time RT-PCR assay

RNA extraction and quantitative real-time RT-PCR assays were carried out according to our previously published protocol [[35], [36], [37], [38], [39]] with modifications. Briefly, 2–4 DRGs were pooled to obtain sufficient RNA. Total RNA was extracted from DRGs using the TRIzol-chloroform method (Invitrogen/Thermo Fisher Scientific, Grand Island, NY) and treated with an excess of DNase I (New England Biolabs, Ipswich, MA) to remove genomic DNA contamination. Complementary DNA (cDNA) was synthesized using ThermoScript Reverse Transcriptase (Invitrogen) with oligo(dT) primers following the manufacturer's protocol. Real-time PCR reactions were prepared in a 20 μl volume containing 1 μl cDNA template, 250 nM forward and reverse primers specific for *Mbnl2* mRNA or *Tuba1a* mRNA (Supplementary Table 1) and 10 μl SsoAdvanced Universal SYBR Green Supermix (Bio-Rad Laboratories). Amplification was performed in a BIO-RAD CFX96 real-time PCR system under the following cycling conditions: an initial denaturation at 95 °C for 3 min, followed by 40 cycles of 95 °C for 10 s, 60 °C for 30 s, and 72 °C for 30 s. *Tuba1α* mRNA was used as the internal reference gene for normalization. Relative MBNL2 mRNA levels were calculated using the 2ˆ-ΔΔCt method.

### *In vitro* RNA stability assay

In vitro RNA stability assay was carried out as described previously [[40], [41], [42]]. CAD cells were cultured in Dulbecco's Modified Eagle's Medium (DMEM; Gibco/ThermoFisher Scientific, Waltham, MA) containing 5% horse serum and 5% v/v fetal bovine serum (FBS; Gibco/ThermoFisher Scientific) at 37 °C in a humidified incubator with 5% CO_2_. Cells were cultured under the indicated AAV5-transduced conditions and treated with Actinomycin D to inhibit transcription (5 μg/ml). The cells were harvested at 0, 3, 6, 9, and 12 h after actinomycin D treatment. Total RNA was extracted using the TRIzol method and *Ccr*2 mRNA level was quantified using quantitative real-time RT-PCR assay as described above.

### RNA immunoprecipitation assay

RNA immunoprecipitation (RIP) was performed using the Magna RIP Kit (Upstate EMD Millipore, Darmstadt, Germany) as described previously [[35], [36], [37], [38], [39]]. Briefly, homogenates from mouse DRGs 14 days after paclitaxel or vehicle injection were suspended in the RIP lysis buffer (Millipore) and incubated overnight at 4 °C with either rabbit anti-MBNL2 antibody or IgG antibody (a negative control). Protein A/G magnetic beads (Thermo Fisher Scientific) were added to capture the antibody-RNA complexes. The immunoprecipitated RNA was purified using TRIzol reagent, reverse-transcribed into cDNA, and subjected to RT-PCR assay using primers specific for *Ccr2* mRNA. Input RNA (total RNA prior to immunoprecipitation) was included as an input control for the comparison with the immunoprecipitated fraction. The distilled H_2_O served as a blank control to exclude the contamination.

### Western blotting analysis

Protein extraction and Western blotting were performed as previously described [[35], [36], [37], [38], [39]] with modifications. Briefly, four DRGs were pooled to obtain sufficient protein. For spinal cord tissues, the lumbar enlargement segments of the spinal cord were quickly dissected, and the dorsal part of the spinal cord was collected. The tissues were homogenized on ice in lysis buffer containing 10 mM Tris, 1 mM phenylmethylsulfonyl fluoride, 5 mM MgCl_2_, 5 mM EGTA, 1 mM EDTA, 1 mM DTT, 40 μM leupeptin, and 250 mM sucrose. Cultured cells were ultrasonicated on ice in the same lysis buffer. The homogenates were centrifuged at 1000×*g* for 15 min at 4 °C. Protein concentrations were measured using the Bio-Rad Protein Assay Kit (Bio-Rad Laboratories). An equal amount of protein (20 μg per sample) was denatured at 99 °C for 5 min, separated on 4–20% SDS-polyacrylamide gels (Bio-Rad Laboratories), and transferred electrophoretically onto polyvinylidene difluoride (PVDF) membranes (Bio-Rad Laboratories). The membranes were blocked with 5% nonfat milk in Tris-buffered saline containing 0.1% Tween-20 (TBS-T) for 1 h at room temperature, followed by overnight incubation at 4 °C with primary antibodies, including rabbit anti-CCR2 (1:1,000; Abcam), rabbit anti-*p*-ERK1/2 (Thr202/Tyr204, 1:800; Cell Signaling Technology), rabbit anti-ERK1/2 (1:800; Cell Signaling Technology), mouse anti-GFAP (1:800; Cell Signaling Technology), rabbit anti-CD68 (1:1,000; Abcam), rabbit anti-IBA1 (1:1,000; Cell Signaling Technology), rabbit anti-MBNL2 (1:500; Invitrogen), and rabbit anti-GAPDH (1:2,000; Santa Cruz). After being washed with TBS-T, the membranes were incubated with horseradish peroxidase (HRP)-conjugated secondary antibodies (anti-rabbit or anti-mouse, 1:5,000; Jackson ImmunoResearch) for 1 h at room temperature. Protein bands were visualized using the Clarity Western ECL Substrate (Bio-Rad) and detected with the ChemiDoc XRS System (Bio-Rad) using Image Lab software. Band intensity was quantified by densitometry using ImageJ software (National Institutes of Health), and all protein bands were normalized to GAPDH (an internal loading control).

### Immunohistochemistry

Mice were deeply anesthetized with isoflurane and transcardially perfused with 25–30 ml of 0.1 M phosphate-buffered saline (PBS, pH 7.4), followed by 30–50 ml of 4% paraformaldehyde in 0.1 M PBS. After perfusion, DRGs were harvested, post-fixed in the same fixative solution for 4–6 h at 4 °C, and cryoprotected in 30% sucrose overnight. Transverse sections (20 μm at thickness) were cut on a cryostat, and every third section was collected from each DRG. After the sections were blocked for 1 h at room temperature in 0.01 M PBS contacting 0.3% Triton X-100 plus 4% goat serum, they were incubated overnight at 4 °C with mouse anti-MBNL2 (1:1,000, Invitrogen) plus chicken anti-β-tubulin III (1:200, EMD Millipore), rabbit anti-glutamine synthetase (GS; 1:500, Sigma-Aldrich), mouse anti-calcitonin gene-related peptide (CGRP; 1:50, Abcam), rabbit anti-NF200 (1:100, Sigma-Aldrich), rabbit anti-CCR2 (1:100) or biotinylated IB4 (1:100, Sigma-Aldrich). After being washed, the sections were incubated with appropriate secondary antibodies conjugated with Cy2 (1:200, Jackson ImmunoResearch Labs, West Grove, PA) or avidin labeled with FITC (1:200, Sigma-Aldrich) for 1 h at room temperature. Fluorescent signals were visualized using a Leica DMI4000 fluorescence microscope equipped with a DFC365 FX camera (Leica).

### Statistical analysis

Statistical analysis was performed using GraphPad Prism 8.5.1 (GraphPad Software, San Diego, CA). Data were presented as means ± standard error (SE). Statistical significance was set at *P* < 0.05. Two-tailed Student's t-tests (paired or unpaired) were used for comparisons between the two groups. For comparisons involving more than two groups, one-way, two-way, or three-way ANOVA was applied as appropriate. When ANOVA showed significant differences, pairwise comparisons between group means were conducted using the post hoc Tukey method.

## Results

### MBNL2 is expressed in mouse peripheral sensory neurons

**Fig. 1:**
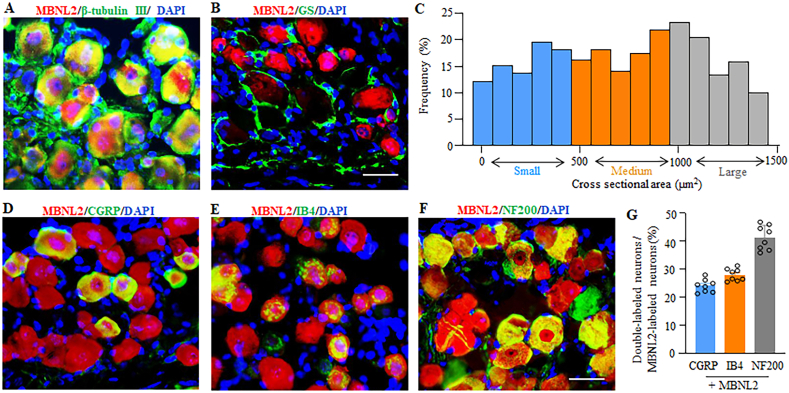
Distribution pattern of MBNL2 in the normal DRG. (A and B) Colocalization of MBNL2 (red) with β-tubulin III (green; A), but not with glutamine synthetase (GS; green; B) in individual cells of mouse DRG. 4′, 6-diamidino-2-phenylindole (DAPI): a nucleus marker. n = 9 sections from 3 mice. Scale bar: 40 μM. (C) Size distribution of MBNL2-positive neuronal soma in normal mouse DRG. The numbers of cells, sections and mice analyzed are the same as in panel A. (D–G) Colocalization of MBNL2 (red) with CGRP (green; D), IB4 (green; E), and NF200 (green; F) in individual DRG neurons. D–H: Representative immunofluorescent images. G: Statistical summary of number of double-labeling neurons. n = 9 sections/marker from 3 mice. Scale bar: 40 μm.

To study the functional relevance of MBNL2 in CINP, we first examined its expression and distribution in the DRG. MBNL2-like immunoreactivity was detected in both the cytoplasm and nuclei of DRG cells (Fig. 1A). MBNL2 was colocalized exclusively with β-tubulin III (a specific neuronal marker) in individual cells of DRG (Fig. 1A). It was undetected in the cells labeled by glutamate synthetase (a marker of satellite cells; Fig. 1B). Approximately 82.4% (742/901) of DRG neurons are positive for MBNL2. Cross-sectional area analysis showed that about 31.54% of MBNL2-labeled neurons were small (<500 μm^2^ in area), 35.18% were medium (500–1000 μm^2^ in area), and 33.28% were large (>1000 μm^2^ in area) (Fig. 1C). Subpopulation analysis further revealed that about 24.1% (139/577) of MBNL2-labeled neurons were positive for CGRP (a marker for small DRG peptidergic neurons; Fig. 1D and G), 28.2% (156/553) were for IB4 (a marker for small non-peptidergic neurons; Fig. 1E and G), and 41.4% (231/558) were for NF200 (a marker for medium/large neurons and myelinated Aβ fibers; Fig. 1F and G). The distribution patterns of MBNL2-positive neurons in the DRG suggest possible involvement of MBNL2 in the transmission and modulation of nociceptive information.

### Systemic administration of paclitaxel downregulates the expression of MBNL2 in DRG neurons

**Fig. 2:**
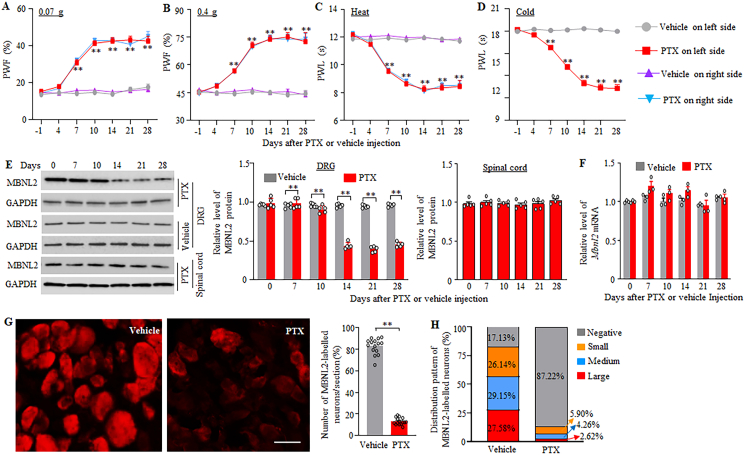
Downregulation of MBNL2 in the DRG after paclitaxel injection. (A–D) Paw withdrawal frequency (PWF) to 0.07 (A) and 0.4 g (B) von Frey filaments and paw withdrawal latency (PWL) to heat (C) and cold (D) stimuli on the indicated days after intraperitoneal injection of paclitaxel (PTX) or vehicle on both left and right sides in male mice. n = 8 mice/group. *∗∗P* < 0.01 versus the vehicle-treated mice at the corresponding day and side by three-way analysis of variance followed by Tukey post hoc test. (E) Level of MBNL2 protein in L3/4 DRGs and spinal cord at the indicated days after intraperitoneal injection of paclitaxel (PTX) or vehicle. n = 5 mice/time point. *∗∗P* < 0.01 by one- or two-way analysis of variance followed by Tukey post hoc test. (F) Level of *Mbnl2* mRNA in L3/4 DRGs at the indicated days after intraperitoneal injection of paclitaxel (PTX) or vehicle. n = 3 mice/time point. Two-way analysis of variance followed by Tukey post hoc test. (G) Number of MBNL2-positive neurons in L3/4 DRGs on day 14 after intraperitoneal injection of paclitaxel (PTX) or vehicle. n = 15 sections (from 5 mice)/group. *∗∗P* < 0.01 by two-tailed, unpaired Student's *t*-test. Scale bar: 40 μm. (d) Changes in the distribution pattern of MBNL2-positive neurons on day 14 after intraperitoneal injection of paclitaxel (PTX) or vehicle. n = 5 mice/group.

We next determined whether MBNL2 expression was altered in the DRG and spinal cord, two major pain-associated regions, after i.p. injection of paclitaxel. Consistent with previous studies [35,36,38,39], i.p. injection of paclitaxel, but not vehicle, produced mechanical allodynia evidenced by significant increases in paw withdrawal frequencies in response to 0.07 and 0.4 g von Frey filaments (Fig. 2A and B) and heat and cold hyperalgesia documented by marked decreases in paw withdrawal latencies in responses to heat and cold stimuli (Fig. 2C and D) on days 7, 10, 14, 21 and 28 post-injection on both ipsilateral and contralateral sides. Unexpectedly, MBNL2 was downregulated in the DRGs on both sides 14, 21 and 28 days, but not 7 and 10 days, post-paclitaxel injection (Fig. 2E). The amounts of MBNL2 protein on 14, 21 and 28 after i.p. paclitaxel decreased by 50%, 52% and 51%, respectively, compared to the corresponding days after i.p. vehicle (Fig. 2E). This downregulation did not occur in the spinal cord during the observation period (Fig. 2E). Interestingly, the levels of *Mbnl2* mRNA were unchanged on days 7, 10, 14, 21 and 28 after i.p. paclitaxel compared to the corresponding days after i.p. vehicle (Fig. 2F). Consistently, only 12.8% (117/915) of DRG neurons were positive for MBNL2 on day 14 post-paclitaxel (Fig. 2G), of which about 5.90% (54/915) of DRG neurons were positive for small, 4.26% (39/915) were medium and 2.62% (24/915) were large (Fig. 2H). Together, these data suggest that paclitaxel-induced downregulation of MBNL2 in DRG neurons may contribute to CINP.

### Rescuing DRG MBNL2 downregulation mitigates the development of paclitaxel-induced CINP

**Fig. 3:**
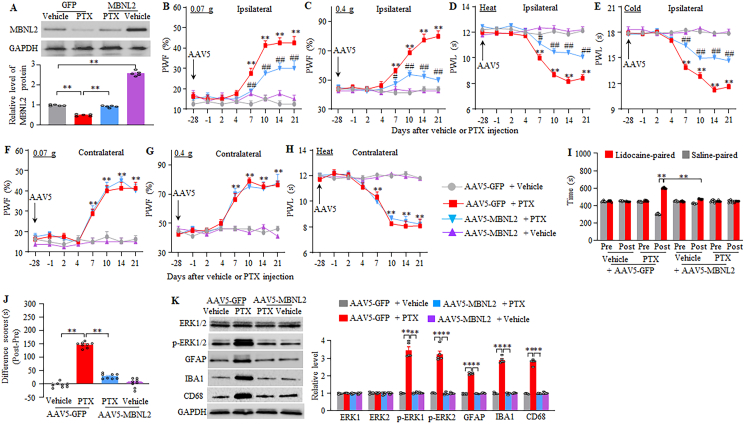
DRG pre-microinjection of AAV5-MBNL2 alleviates the development of paclitaxel-induced neuropathic pain and activations of spinal cord cells in male mice. (A) Level of MBNL2 protein in the microinjected L3/4 DRGs on day 21 after paclitaxel (PTX) or vehicle injection in male mice with DRG pre-microinjection of AAV5-MBNL2 or AAV5-GFP for 28 days. n = 5 mice/group. *∗∗P* < 0.01 by two-way analysis of variance followed by Tukey post hoc test. (B–H) Paw withdrawal frequency (PWF) to 0.07 (B and F) and 0.4 g (C and G) von Frey filaments and paw withdrawal latency (PWL) to heat (D and H) and cold (E) stimuli on the indicated days after intraperitoneal injection of paclitaxel (PTX) or vehicle on the ipsilateral (B–E) and contralateral (F–H) sides in male mice with DRG pre-microinjection of AAV5-MBNL2 or AAV5-GFP for 28 days. n = 8 mice/group. *∗∗P* < 0.01 versus the AAV5-GFP plus vehicle-treated mice on the corresponding day. *#P* < 0.05, *##P* < 0.01 versus the AAV5-GFP plus paclitaxel-treated mice on the corresponding day. Three-way analysis of variance followed by Tukey post hoc test. (I and J) Ongoing nociceptive responses as assessed by the conditional place preference paradigm on day 14 post-paclitaxel or vehicle in male mice with DRG pre-microinjection of AAV5-MBNL2 or AAV5-GFP for 28 days. n = 8 mice/group. *∗∗P* < 0.01 by two (J)- or three (I)-way analysis of variance followed by Tukey post hoc test. (K) Levels of phosphorylated ERK1/2 (*p*-ERK1/2), total ERK1/2, GFAP, IBA1, and CD68 in the ipsilateral L3/4 spinal cord dorsal horn 21 days after paclitaxel (PTX) or vehicle injection in male mice with DRG pre-microinjection of AAV5-MBNL2 or AAV5-GFP for 28 days. n = 5 mice/group. *∗∗P* < 0.01 by two-way analysis of variance followed by Tukey post hoc test.

To investigate whether MBNL2 downregulation in the DRG contributes to the development of CINP, we restored DRG MBNL2 expression by microinjecting adeno-associated virus serotype 5 expressing full-length *Mbnl2* mRNA (AAV5-MBNL2) into the unilateral L3/4 DRGs 28 days before i.p. injection of paclitaxel or vehicle in male mice, because AAV5 takes 3–4 weeks for robustly expressing the target gene [43,44]. Mice microinjected with AAV5 expressing GFP (AAV5-GFP) served as the controls. As expected, the level of MBNL2 in the AAV5-GFP-microinjected L3/4 DRGs 21 days post-paclitaxel was markedly reduced by 50% compared to the corresponding AAV5-GFP-microinjected vehicle group (Fig. 3A). This reduction was completely reversed in the AAV5-MBNL2-microinjected L3/4 DRGs 21 days post-paclitaxel (Fig. 3A). Additionally, on day 21 post-vehicle, the level of MBNL2 in the AAV5-MBNL2-microinjected L3/4 DRGs was increased by 2.61-fold compared with the AAV5-GFP-microinjected vehicle group (Fig. 3A).

Consistent with the behavioral observations above, mechanical allodynia and heat and cold hyperalgesia were detected on both ipsilateral and contralateral sides from days 7–21 post-paclitaxel in AAV5-GFP-microinjected male mice (Fig. 3B–H). DRG microinjection of AAV5-MBNL2 substantially mitigated the paclitaxel-induced increases in paw withdrawal frequencies to 0.07 g and 0.4 g von Frey filament stimuli and the paclitaxel-induced decreases in paw withdrawal latencies to heat and cold stimuli from days 7–21 post-paclitaxel on the ipsilateral, but not contralateral, side (Fig. 3B–H). Neither AAV5-MBNL2 nor AAV5-GFP microinjection altered basal paw withdrawal responses on either side of the vehicle-treated male mice (Fig. 3B–H). All microinjected male mice displayed normal locomotor activities (Supplementary Table 2). Furthermore, DRG microinjection of AAV5-MBNL2, but not AAV5-GFP, attenuated the spontaneous pain in male mice, as documented by a significant decrease in preference for the lidocaine-paired chamber on day 14 post-paclitaxel (Fig. 3I–J). Vehicle-treated male mice microinjected with either virus revealed no marked preference for either chamber (Fig. 3I–J).

Paclitaxel injection can activate neurons, glial cells, and macrophages in the spinal dorsal horn either through its direct action in the spinal dorsal horn or through DRG neuronal hyperexcitability–induced increases in the release of neurotransmitters and neuromodulators from primary afferents [37,38]. To further validate the behavioral responses observed above, we assessed the effect of DRG microinjection of AAV5-MBNL2 on the activities of neurons, glial cells and microphages in the spinal dorsal horn following paclitaxel injection. In line with previous studies [37,38], the levels of *p*-ERK1/2 (a marker for neuronal hyperactivity), GFAP (a marker for astrocyte), Iba1 (a marker for microglia) and CD68 (a marker for macrophages/monocytes), but not total ERK1/2, were markedly increased in the L3/4 dorsal horn of AAV5-GFP-microinjected male mice on day 21 post-paclitaxel (Fig. 3K). These increases were absent in the ipsilateral L3/4 dorsal horn of AAV5-MBNL2-microinjected male mice 21 days post-paclitaxel (Fig. 3K). Neither virus changed basal levels of these markers in the dorsal horn of vehicle-treated male mice (Fig. 3K). These results suggest that rescuing MBNL2 expression in the DRGs alleviates central sensitization of the dorsal horn under the conditions of CINP.

**Fig. 4:**
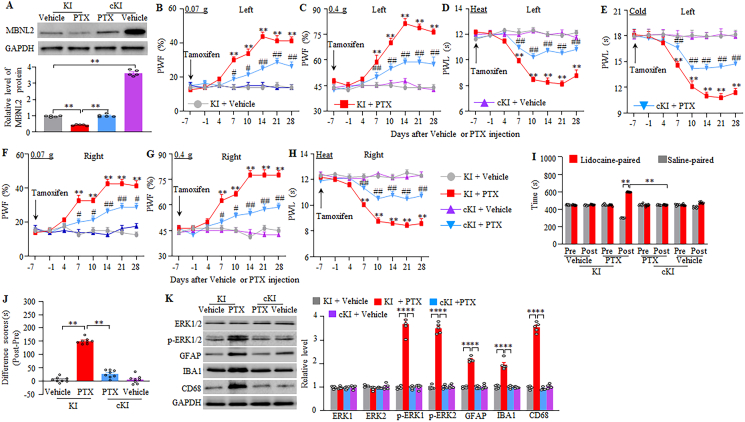
Tamoxifen injection in inducible conditional MBNL2 knock-in (cKI) male mice, but not in MBNL2 knock-in (KI) male mice, mitigates the development of paclitaxel-induced neuropathic pain and activations of spinal cord cells. (A) Level of MBNL2 protein in L3/4 DRGs on day 28 after paclitaxel (PTX) or vehicle injection in male KI or cKI mice with pre-injection of tamoxifen daily for 7 days. n = 5 mice/group. *∗∗P* < 0.01 by two-way analysis of variance followed by Tukey post hoc test. (B–H) Paw withdrawal frequency (PWF) to 0.07 (B and F) and 0.4 g (C and G) von Frey filaments and paw withdrawal latency (PWL) to heat (D and H) and cold (E) stimuli on the indicated days after intraperitoneal injection of paclitaxel (PTX) or vehicle on the left (B–E) and right (F–H) sides in male KI or cKI mice with pre-injection of tamoxifen daily for 7 days. n = 8 mice/group. *∗∗P* < 0.01 versus the vehicle-treated KI mice on the corresponding day. *#P* < 0.05, *##P* < 0.01 versus the paclitaxel-treated KI mice on the corresponding day. Three-way analysis of variance followed by Tukey post hoc test. (I and J) Ongoing nociceptive responses as assessed by the conditional place preference paradigm on day 21 post-paclitaxel or vehicle in male KI or cKI mice with DRG pre-injection of tamoxifen daily for 7 days. n = 8 mice/group. *∗∗P* < 0.01 by two (J)- or three (I)-way analysis of variance followed by Tukey post hoc test. (K) Levels of phosphorylated ERK1/2 (*p*-ERK1/2), total ERK1/2, GFAP, IBA1, and CD68 in L3/4 spinal cord dorsal horn 28 days after paclitaxel (PTX) or vehicle injection in male KI or cKI mice with pre-injection of tamoxifen daily for 7 days. n = 5 mice/group. *∗∗P* < 0.01 by two-way analysis of variance followed by Tukey post hoc test.

**Fig. 5:**
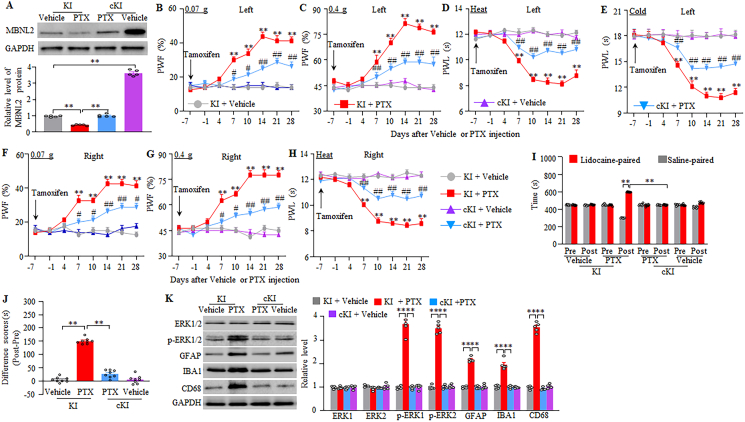
Tamoxifen injection in inducible conditional MBNL2 knock-in (cKI) female mice, but not in MBNL2 knock-in (KI) female mice, mitigates the development of paclitaxel-induced neuropathic pain and activations of spinal cord cells. (A) Level of MBNL2 protein in L3/4 DRGs on day 28 after paclitaxel (PTX) or vehicle injection in female KI or cKI mice with pre-injection of tamoxifen daily for 7 days. n = 5 mice/group. *∗∗P* < 0.01 by two-way analysis of variance followed by Tukey post hoc test. (B–H) Paw withdrawal frequency (PWF) to 0.07 (B and F) and 0.4 g (C and G) von Frey filaments and paw withdrawal latency (PWL) to heat (D and H) and cold (E) stimuli on the indicated days after intraperitoneal injection of paclitaxel (PTX) or vehicle on the left (B–E) and right (F–H) sides in female KI or cKI mice with pre-injection of tamoxifen daily for 7 days. n = 8 mice/group. *∗∗P* < 0.01 versus the vehicle-treated KI mice on the corresponding day. *#P* < 0.05, *##P* < 0.01 versus the paclitaxel-treated KI mice on the corresponding day. Three-way analysis of variance followed by Tukey post hoc test. (I and J) Ongoing nociceptive responses as assessed by the conditional place preference paradigm on day 21 post-paclitaxel or vehicle in female KI or cKI mice with DRG pre-injection of tamoxifen daily for 7 days. n = 8 mice/group. *∗∗P* < 0.01 by two (J)- or three (I)-way analysis of variance followed by Tukey post hoc test. (K) Levels of phosphorylated ERK1/2 (*p*-ERK1/2), total ERK1/2, GFAP, IBA1, and CD68 in L3/4 spinal cord dorsal horn 28 days after paclitaxel (PTX) or vehicle injection in female KI or cKI mice with pre-injection of tamoxifen daily for 7 days. n = 5 mice/group. *∗∗P* < 0.01 by two-way analysis of variance followed by Tukey post hoc test.

To exclude the possibility that the observed phenotypic changes above may be associated with microinjection-caused tissue damage, we generated the *Mbnl2* knock-in (KI) mice in which full-length *Mbnl2* cDNA was inserted into the ROSA26 allele and could be Cre-dependent activated under the control of the CAG promoter. To rescue MBNL2 expression in the DRG neurons, we crossed the KI mice with sensory neuron-specific *Advillin*^*Cre-ERT2*^ mice to generate the inducible conditional *Mbnl2* KI (cKI) mice, in which i.p. injection of tamoxifen at 1 mg daily for 7 days before paclitaxel injection rescued MBNL2 expression 28 days post-paclitaxel (Fig. 4A). This rescue also impaired mechanical allodynia and heat/cold hyperalgesia on both sides from days 7–28 post-paclitaxel (Fig. 4B–H), attenuated spontaneous pain on day 21 post-paclitaxel (Fig. 4I–J) and abolished the activations of dorsal horn neurons, astrocytes, microglia cells and macrophages on day 28 post-paclitaxel (Fig. 4K) in male mice. As expected, basal paw responses and dorsal horn cell activities on both sides of vehicle-treated male KI mice or cKI mice were not changed (Fig. 4B–K), although a marked increase of MBNL2 in the DRGs of the tamoxifen-injected and vehicle-treated male cKI mice (Fig. 4A). Similar results were observed in the paclitaxel- or vehicle-treated female KI mice and cKI mice with pre-injection of tamoxifen (Fig. 5A–K). All tamoxifen-treated KI mice or cKI mice with i.p. injection of paclitaxel or vehicle exhibited normal locomotor activity (Supplementary Table 2).

Collectively, these findings strongly support a role for DRG MBNL2 downregulation in the pathogenesis of CINP.

### Rescuing DRG MBNL2 downregulation attenuates the maintenance of paclitaxel-induced CINP

**Fig. 6:**
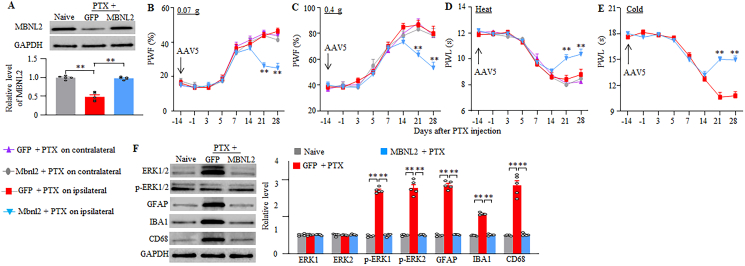
DRG pre-microinjection of AAV5-MBNL2 alleviates the maintenance of paclitaxel-induced neuropathic pain and activations of spinal cord cells in male mice. (A) Level of MBNL2 protein in the ipsilateral L3/4 DRGs in naïve male mice or on day 28 after paclitaxel (PTX) injection in male mice with DRG pre-microinjection of AAV5-MBNL2 (MBNL2) or AAV5-GFP (GFP) for 14 days. n = 5 mice/group. *∗∗P* < 0.01 by one-way analysis of variance followed by Tukey post hoc test. (B–E) Paw withdrawal frequency (PWF) to 0.07 (B) and 0.4 g (C) von Frey filaments and paw withdrawal latency (PWL) to heat (D) and cold (E) stimuli on the indicated days after intraperitoneal injection of paclitaxel (PTX) or vehicle on the ipsilateral and contralateral sides in male mice with DRG pre-microinjection of AAV5-MBNL2 (MBNL2) or AAV5-GFP (GFP) for 14 days. n = 8 mice/group. *∗∗P* < 0.01 versus the corresponding basal responses on day −14 post-paclitaxel. *##P* < 0.01 versus the AAV5-GFP plus paclitaxel-treated male mice on the corresponding day on the ipsilateral side. Three-way analysis of variance followed by Tukey post hoc test. (F) Levels of phosphorylated ERK1/2 (*p*-ERK1/2), total ERK1/2, GFAP, IBA1, and CD68 in the ipsilateral L3/4 spinal cord dorsal horn in naïve male mice or 28 days after paclitaxel (PTX) injection in male mice with DRG pre-microinjection of AAV5-MBNL2 or AAV5-GFP for 14 days. n = 5 mice/group. *∗∗P* < 0.01 by one-way analysis of variance followed by Tukey post hoc test.

The role of DRG MBNL2 downregulation in maintening paclitaxel-induced CINP was also examined. AAV5 was microinjected into unilateral L3/4 DRGs 14 days before i.p. injection of paclitaxel or vehicle. As expected, the reduction in the level of MBNL2 in the ipsilateral L3/4 DRGs 28 days post-paclitaxel in the AAV5-GFP-microinjected group was fully rescued in the AAV5-MBNL2-microinjected group (Fig. 6A). Mechanical allodynia and heat/cold hyperalgesia were fully developed on the ipsilateral and contralateral sides on days 7 and 14 post-paclitaxel in both AAV5-MBNL2-and AAV5-GFP-microinjected male mice (Fig. 6B–E). These nociceptive hypersensitivities were attenuated only on the ipsilateral side on days 21 and 28 post-paclitaxel in the AAV5-MBNL2- (but not AAV5-GFP-) microinjected male mice (Fig. 6B–E). Consistently, activations of neurons, astrocytes, microglia cells and microphages in the ipsilateral L3/4 dorsal horn on day 28 post-paclitaxel from the AAV5-GFP-microinjected group were absent in the AAV5-MBNL2-microinjected group (Fig. 6F). Together, these data indicate that DRG MBNL2 downregulation contributes to the maintenance of CINP.

### Mimicking paclitaxel-induced DRG MBNL2 downregulation produces nociceptive hypersensitivity

**Fig. 7:**
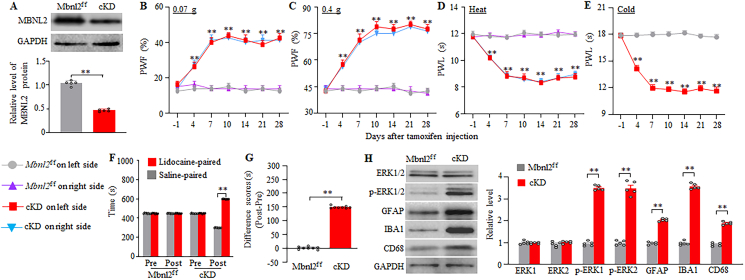
MBNL2 knockdown in DRG neurons produces neuropathic pain-like symptoms and activations of spinal cord cells in male mice. (A) Level of MBNL2 protein in the L3/4 DRGs in male *Mbnl2*^f/f^ mice and inducible conditional MBNL2 knockdown (cKD) mice on day 28 after tamoxifen injection. n = 5 mice/group. *∗∗P* < 0.01 by two-tailed, unpaired Student's *t*-test. (B–E) Paw withdrawal frequency (PWF) to 0.07 (B) and 0.4 g (C) von Frey filaments and paw withdrawal latency (PWL) to heat (D) and cold (E) stimuli on the indicated days after intraperitoneal injection of tamoxifen on both left and right sides in male mice. n = 8 mice/group. *∗∗P* < 0.01 versus the tamoxifen-treated *Mbnl2*^f/f^ mice on the corresponding day and side by three-way analysis of variance followed by Tukey post hoc test. (F and G) Ongoing nociceptive responses as assessed by the conditional place preference paradigm on day 21 post-tamoxifen in male *Mbnl2*^f/f^ mice and cKD mice. n = 8 mice/group. *∗∗P* < 0.01 by two-way analysis of variance followed by Tukey post hoc test (F) or two-tailed, unpaired Student's *t*-test (G). (H) Levels of phosphorylated ERK1/2 (*p*-ERK1/2), total ERK1/2, GFAP, IBA1, and CD68 in L3/4 spinal cord dorsal horn 28 days after tamoxifen injection in male *Mbnl2*^f/f^ mice and cKD mice. n = 5 mice/group. *∗∗P* < 0.01 by two-tailed, unpaired Student's *t*-test.

**Fig. 8:**
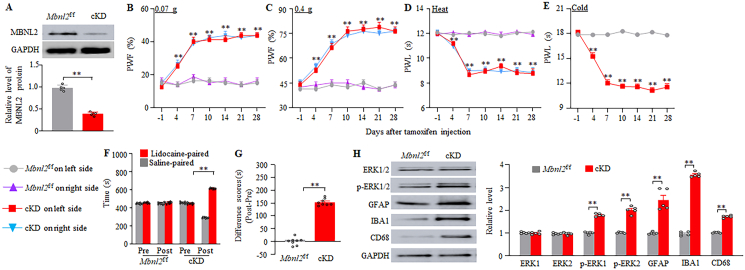
MBNL2 knockdown in DRG neurons produces neuropathic pain-like symptoms and activations of spinal cord cells in female mice. (A) Level of MBNL2 protein in the L3/4 DRGs in female *Mbnl2*^f/f^ mice and inducible conditional MBNL2 knockdown (cKD) mice on day 28 after tamoxifen injection. n = 5 mice/group. *∗∗P* < 0.01 by two-tailed, unpaired Student's *t*-test. (B–E) Paw withdrawal frequency (PWF) to 0.07 (B) and 0.4 g (C) von Frey filaments and paw withdrawal latency (PWL) to heat (D) and cold (E) stimuli on the indicated days after intraperitoneal injection of tamoxifen on both left and right sides in female mice. n = 8 mice/group. *∗∗P* < 0.01 versus the tamoxifen-treated *Mbnl2*^f/f^ mice on the corresponding day and side by three-way analysis of variance followed by Tukey post hoc test. (F and G) Ongoing nociceptive responses as assessed by the conditional place preference paradigm on day 21 post-tamoxifen in female *Mbnl2*^f/f^ mice and cKD mice. n = 8 mice/group. *∗∗P* < 0.01 by two-way analysis of variance followed by Tukey post hoc test (F) or two-tailed, unpaired Student's *t*-test (G). (H) Levels of phosphorylated ERK1/2 (*p*-ERK1/2), total ERK1/2, GFAP, IBA1, and CD68 in L3/4 spinal cord dorsal horn 28 days after tamoxifen injection in female *Mbnl2*^f/f^ mice and cKD mice. n = 5 mice/group. *∗∗P* < 0.01 by two-tailed, unpaired Student's *t*-test.

We next asked whether DRG MBNL2 downregulation is sufficient to induce CINP-like phenotypes. To this end, we knocked down MBNL2 in the DRG neurons by i.p. injection of tamoxifen (at 1 mg daily for 7 days) in male inducible conditional MBNL2 knockdown (cKD) mice, which were generated by crossing *Mbnl2*^*f/f*^ mice with sensory neuron-specific *Advillin*^*cre-ERT2*^ mice. As expected, the level of MBNL2 in the DRG of male cKD mice on day 28 after the first injection of tamoxifen was reduced by 50% compared to the tamoxifen-injected male *Mbnl2*^*f/f*^ mice (Fig. 7A). Importantly, the tamoxifen-injected male cKD mice displayed significant increases in paw withdrawal frequencies to 0.07 and 0.4 g von Frey filament stimuli and decreases in paw withdrawal latencies to heat and cold stimuli on both ipsilateral and contralateral sides from days 4–28 (at least) after the first injection of tamoxifen (Fig. 7B–E), a substantial increase in preference for the lidocaine-paired chamber on day 21 after the first injection of tamoxifen (Fig. 7F–G), and marked elevations in the levels of *p*-ERK1/2 (but not total ERK1/2), GFAP, IBA1 and CD68 in bilateral L3/4 dorsal horn on day 28 after the first injection of tamoxifen (Fig. 7H). No locomotor abnormalities were observed in both tamoxifen-treated male *Mbnl2*^*f/f*^ mice and cKD mice (Supplementary Table 2). Similar observations were found in female *Mbnl2*^*f/f*^ mice and cKD mice after tamoxifen injection (Fig. 8A–H; Supplementary Table 2). Taken together, these findings indicate that DRG MBNL2 downregulation is sufficient to produce CINP-like symptoms even in the absence of paclitaxel treatment.

### MBNL2 downregulation participates in the paclitaxel-induced CCR2 increase in the DRG neurons

**Fig. 9:**
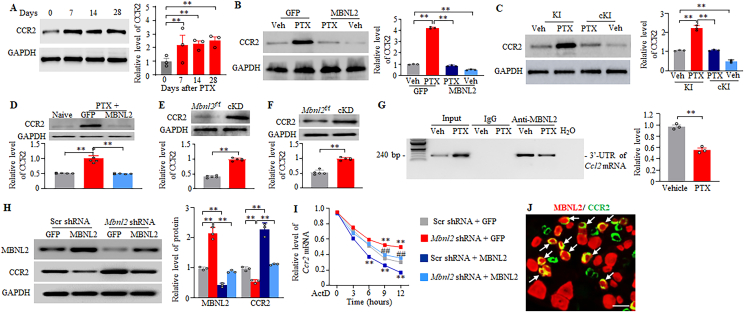
MBNL2 downregulation contributes to the paclitaxel-induced increase of CCR2 in the DRG neurons. (A) Level of CCR2 in L3/4 DRGs at the indicated days after intraperitoneal injection of paclitaxel (PTX). n = 3 mice/time point. *∗∗P* < 0.01 by one-way analysis of variance followed by Tukey post hoc test. (B) Level of CCR2 in the ipsilateral L3/4 DRGs 21 days after intraperitoneal injection of paclitaxel (PTX) or vehicle in male mice with DRG pre-microinjection of AAV5-GFP or AAV5-MBNL2 for 28 days. n = 3 mice/group. *∗∗P* < 0.01 by two-way analysis of variance followed by Tukey post hoc test. (C) Level of CCR2 in L3/4 DRGs on day 28 after paclitaxel (PTX) or vehicle injection in male KI or cKI mice with pre-injection of tamoxifen daily for 7 days. n = 3 mice/group. *∗∗P* < 0.01 by two-way analysis of variance followed by Tukey post hoc test. (D) Level of CCR2 in the ipsilateral L3/4 DRGs 28 days after intraperitoneal injection of paclitaxel (PTX) in male mice with DRG pre-microinjection of AAV5-GFP or AAV5-MBNL2 for 14 days. n = 5 mice/group. *∗∗P* < 0.01 by one-way analysis of variance followed by Tukey post hoc test. (E and F) Level of CCR2 in the L3/4 DRGs from male (D) and female (E) *Mbnl2*^f/f^ mice and inducible conditional MBNL2 knockdown (cKD) mice on day 28 after tamoxifen injection. n = 5 mice/group. *∗∗P* < 0.01 by two-tailed, unpaired Student's *t*-test. (G) Binding activity of MBNL2 to 3′-UTR of *Ccr2* mRNA assessed using RNA immunoprecipitation analysis in the L3/4 DRGs 14 days post-paclitaxel or vehicle in male mice. n = 3 biological repeats (mice)/group. *P* < 0.01 by two-tailed, unpaired Student's *t*-test. (H and I) *Ccr2* mRNA stability in CAD cells transduced by AAV5 as shown and treated with actinomycin D (ActD) for the indicated times. Scr shRNA: AAV5-scarmbled shRNA. GFP: AAV5-GFP. *Mbnl2* shRNA: AAV5-*Mbnl2* shRNA. MBNL2: AAV5-MBNL2. n = 3 biological repeats/group or time point. H: Levels of MBNL1 and CCR2 12 h after ActD treatment in CAD cells treated as indicated. ∗∗*P* < 0.01 by one-way analysis of variance followed by Tukey post hoc test. I: Level of *Ccr2* mRNA in CAD cells treated as indicated at the different time points after ActD treatment. ∗∗*P* < 0.01 versus the Scr shRNA plus GFP group at the corresponding time points and ##*P* < 0.01 versus the *Mbnl2* shRNA plus GFP group at the corresponding time points by one-way analysis of variance followed by Tukey post hoc test. (J) Colocalization of MBNL2 (red) with CCR2 (green) in the individual DRG neurons (arrows) of naïve mice. n = 3 mice. Scale bar: 40 μm.

We finally explored how DRG MBNL2 downregulation is responsible for CINP. Using the online database ATtRACT (http://attract.cnic.es), we identified several mRNA candidates, including chemokine C–C motif receptor 2 (*Ccr2*) mRNA, as potential binding partners for MBNL2. Considering that DRG increased CCR2 contributes to the development and maintenance of CINP [13,14,26], we proposed that MBNL2 downregulation participates in the paclitaxel-induced increase of CCR2 in the DRG. Consistent with previous studies [13,14], the level of CCR2 was significantly and time-dependently increased in the DRGs on both sides 7, 14 and 28 days after i.p. injection of paclitaxel (Fig. 9A). The CCR2 increases in the ipsilateral L3/4 DRGs 21 days after paclitaxel injection in the mice pre-microinjected with AAV5-GFP for 28 days and 28 days after paclitaxel injection in the KI mice pre-injected with tamoxifen for 7 days were not observed in AAV5-MBNL2–pre-microinjected and paclitaxel treated mice (Fig. 9B) and in tamoxifen-pre-injected and paclitaxel-treated cKI mice (Fig. 9C), respectively. Similarly, the CCR2 increase in the ipsilateral L3/4 DRGs 28 days after paclitaxel injection in the mice pre-microinjected with AAV5-GFP for 14 days was not detected in the AAV5-MBNL2-pre-microinjected and paclitaxel-treated mice (Fig. 9D). Basal level of CCR2 was markedly reduced in the ipsilateral L3/4 DRGs of AAV5-MBNL2-pre-microinjected mice 21 days post-vehicle (Fig. 9B) or in the DRGs of tamoxifen-pre-injected cKI mice 28 days post-vehicle (Fig. 9C). Conversely, the amounts of CCR2 were significantly elevated in the DRGs of male and female MBNL2 cKD mice 28 days after tamoxifen injection (Fig. 9E and F). More importantly, our RIP assay showed the binding of MBNL2 to the 3′-untranslated region (UTR) of *Ccr2* mRNA in the L3/4 DRGs of male mice 14 days after vehicle injection (Fig. 9G)*.* This binding was significantly decreased 14 days post-paclitaxel injection (Fig. 9G). This decrease is attributed to a reduction in the level of MBNL2 (Fig. 2E), rather than an increase of *Ccr2* mRNA (Input in Fig. 9G), in the L3/4 DRGs 14 days post-paclitaxel injection. Furthermore, after transcriptional inhibition with Actinomycin D, the decay rate of *Ccr2* mRNA was significantly slower after MBNL2 knockdown in AAV5-*Mbnl2* shRNA– plus AAV5-GFP–transduced cultured CAD cells than in control AAV5-GFP– plus AAV5-Scr siRNA–transduced cultured CAD cells (Fig. 9H and I), resulting in the increases of *Ccr2* mRNA and CCR2 protein (Fig. 9H and I). These effects were abolished after MBNL2 knockdown was rescued in AAV5-MBNL2-plus AAV5-*Mbnl2* shRNA-transduced CAD cells (Fig. 9H and I). As expected, the decay rate of *Ccr2* mRNA was significantly increased after MBNL2 overexpression in AAV5-MBNL2– plus AAV5-Scr siRNA–transduced cultured CAD cells than in AAV5-GFP– plus AAV5-Scr siRNA–transduced cultured CAD cells (Fig. 9H and I), resulting in significantly lower levels of *Ccr2* mRNA and CCR2 protein (Fig. 9H and I). These findings indicate that MBNL2 negatively regulates the expression of CCR2 in the DRG under CINP conditions. Given that MBNL2 and CCR2 are co-expressed in the same individual DRG neurons (Fig. 9J), DRG MBNL2 downregulation is responsible for CINP through upregulating CCR2 in the DRG neurons.

## Discussion

Paclitaxel is a widely used chemotherapy drug in the clinic. Nociceptive hypersensitivities caused by systemic injection of paclitaxel in mice mimic CINP in clinical settings. Understanding how paclitaxel injection produces nociceptive hypersensitivity may open new avenues for managing CINP. The present study demonstrated the time-dependent downregulation of MBNL2 in the DRG neurons following i.p. injection of paclitaxel. Rescuing this downregulation attenuated the paclitaxel-induced increase of DRG CCR2 and the development and maintenance of nociceptive hypersensitivity. Mimicking this downregulation elevated DRG CCR2 expression and augmented the responses to mechanical, heat and cold stimuli in naïve mice. Our findings indicate that MBNL2 downregulation contributes to CINP likely through elevating CCR2 expression in the DRG neurons.

Like other RNA-binding proteins including SYNCRIP [45], RALY [46] and FUS [47], MBNL2 appears to be dynamically regulated in DRG neurons under the conditions of neuropathic pain. Consistent with previous reports [29,30], MBNL2 was expressed exclusively in the DRG neurons and detected in both cytoplasm and nuclei of these neurons, indicating that MBNL2 may have distinct functions in these two neuronal compartments. The present study demonstrated that systemic administration of paclitaxel substantially downregulated the expression of MBNL2 protein in the DRG on days 14, 21 and 28 post-paclitaxel, but not on days 7 and 10 post-paclitaxel administration. Unexpectedly, *Mbnl2* mRNA expression was not altered in the DRG after systemic paclitaxel injection during the observation period. These results suggest that systemic administration of paclitaxel alters post-transcriptional and/or post-translational regulation of MBNL2 in the late phase of CINP. Although the precise mechanisms underlying this alteration are still unclear in the present study, possible involvements of increased protein degradation, reduced translation efficiency, ubiquitination and increased protein turnover cannot be excluded and remain to be investigated in our future research.

To define the role of DRG MBNL2 downregulation in the CINP, we employed both DRG AAV5-MBNL2 microinjection and sensory neuron-specific MBNL2 inducible cKI or cKD transgenic mice. We demonstrated that either the microinjection of AAV5-MBNL2 into the unilateral L3/4 DRGs or the i.p. injection of tamoxifen in the MBNL2 cKI mice rescued the paclitaxel-induced DRG MBNL2 downregulation and attenuated the paclitaxel-induced nociceptive hypersensitivity without altering basal (acute) pain and locomotor functions. These findings indicate the specificity and selectivity of the effects of both strategies. AAV5-MBNL2 microinjection directly rescued the expression of MBNL2 in small, medium and large neurons of the microinjected L3/4 DRGs from the paclitaxel-treated mice, as CMV-controlled AAV5 transduces into these three types of neurons, but not other DRG cells [43,44,48,49]. However, AAV5 microinjection may cause potential damage to the DRG, which interferes with interpreting the observed phenotypic changes. In contrast, DRG MBNL2 overexpression in MBNL2 cKI mice after i.p. injection of tamoxifen occurs exclusively in sensory neurons, but this overexpression is seen in all DRGs and trigeminal ganglia. Thus, the data from these two strategies are complementary. More importantly, DRG overexpression or knockdown of MBNL2 did not affect normal locomotor activity, suggesting that MBNL2 expressed in medium and large DRG neurons does not contribute to proprioceptive functions. Overall, these findings suggest that targeting DRG MBNL2 may have therapeutic potential, although further studies are required to evaluate its safety and possible side effects.

The downregulated DRG MBNL2 contributes to the paclitaxel-induced CINP likely through elevating the expression of CCR2 in the DRG neurons. CCR2 activation directly excites small, medium and large neurons in the DRG [[15], [16], [17], [18]]. CCL2 sensitizes nociceptors through the CCR2-mediated transactivation of TRPV1 or Nav1.8 [15,[19], [20], [21]]. Systemic administration of paclitaxel or oxaliplatin increased the expression of DRG CCR2 [13,14]. Inhibition or knockdown of DRG CCR2 alleviated CINP [13,14,26]. DRG increased CCR2 is likely critical for paclitaxel-induced ectopic discharges in DRG neurons and CINP. This conclusion is supported indirectly by our behavioral observations showing that rescuing DRG MBNL2 downregulation not only blocked the paclitaxel-induced CCR2 increase in the DRG neurons but also alleviated both development and maintenance of the paclitaxel-induced nociceptive hypersensitivity, although MBNL2 expression remained unchanged on days 7 and 10 following paclitaxel administration. The antinociceptive effect of DRG overexpression of MBNL during the CINP development period may be attributed to the blockage of paclitaxel-induced increase of DRG CCR2, because DRG overexpression of MBNL2 reduced basal CCR2 level in the DRG from the vehicle-treated mice and CCR2 was increased starting from day 7 post-paclitaxel injection [13,14]. Considering that DRG MBNL2 knockdown elevated the CCR2 expression in the DRG and produced CINP-like symptoms, MBNL2 mitigates CINP likely through blocking CCR2 expression in DRG neurons. Our *in vitro* RNA stability assay revealed that MBNL2 knockdown slowed the decay rate of *Ccr2* mRNA, an effect that was abolished upon rescue of MBNL2 expression. Conversely, MBNL2 overexpression accelerated the decay rate of *Ccr2* mRNA. Given that MBNL2 binds to *Ccr2* 3′UTR in the DRG, MBNL2 likely destabilizes the expression of *Ccr2* mRNA expression possibly through recruiting RNA decay machinery or altering RNA–protein interactions [30,50]. Other potential mechanisms including alternative polyadenylation [51], interaction with microRNA pathways [52] and coupling RNA splicing with mRNA turnover [30] cannot be excluded in MBNL2-mediated negative regulation of *Ccr2* mRNA. A reduction in the binding of MBNL2 to 3′-UTR of *Ccr2* mRNA in the DRG following paclitaxel injection strongly suggests that DRG MBNL2 downregulation contributes to the paclitaxel-caused CINP likely by enhancing *Ccr2* mRNA stabilization, leading to the elevations of *Ccr2* mRNA and CCR2 protein in the DRG. Besides CCR2, the functions of other downstream targets in mediating the role of downregulated MBNL2 in the paclitaxel-induced CINP cannot be ruled out. Notably, MBNL2 is also expressed in the nucleus of DRG neurons. Whether nucleolar MBNL2 contributes to CINP by regulating alternative splicing through its interaction with pre-mRNA [30] in the DRG remains to be determined.

In conclusion, the present study demonstrated that DRG overexpression of MBNL2 through DRG microinjection of AAV5-MBNL2 alleviated the development and maintenance of paclitaxel-induced CINP, without affecting acute/basal pain and locomotor activity. Given that AAV has recently been FDA-approved as a gene therapy strategy for use in humans [[53], [54], [55], [56]], AAV5-MBNL2 may be used as a potential pain killer in CINP treatment. In addition, further work identifying druggable downstream pathways or small-molecule modulators of MBNL2 function would substantially enhance translational relevance. Nevertheless, potential adverse effects will need to be carefully evaluated because MBNL2 is widely expressed in multiple tissues.

## Author contributions

Study conception and design: KY, RM, YXT.

Data acquisition and analysis: KY, RM, BW, XF, XL, XM, HS, BMK, MSS, HH, YXT.

Drafting figures: KY, RM, BW, YXT.

Drafting manuscript: KY, YXT.

Finalizing manuscript: YXT.

## Declaration of competing interests

X The authors declare that they have no known competing financial interests or personal relationships that could have appeared to influence the work reported in this paper.

## References

[1] Grisold W., Cavaletti G., Windebank A.J. (2012). Peripheral neuropathies from chemotherapeutics and targeted agents: diagnosis, treatment, and prevention. Neuro Oncol.

[2] Windebank A.J., Grisold W. (2008). Chemotherapy-induced neuropathy. J Peripher Nerv Syst.

[3] Argyriou A.A., Bruna J., Marmiroli P., Cavaletti G. (2012). Chemotherapy-induced peripheral neurotoxicity (CIPN): an update. Crit Rev Oncol Hematol.

[4] Devor M. (1999). Unexplained peculiarities of the dorsal root ganglion. Pain.

[5] Jimenez-Andrade J.M., Herrera M.B., Ghilardi J.R., Vardanyan M., Melemedjian O.K., Mantyh P.W. (2008). Vascularization of the dorsal root ganglia and peripheral nerve of the mouse: implications for chemical-induced peripheral sensory neuropathies. Mol Pain.

[6] Li Y., North R.Y., Rhines L.D., Tatsui C.E., Rao G., Edwards D.D. (2018). DRG voltage-gated sodium channel 1.7 is upregulated in paclitaxel-induced neuropathy in rats and in humans with neuropathic pain. J Neurosci.

[7] Li Y., Tatsui C.E., Rhines L.D., North R.Y., Harrison D.S., Cassidy R.M. (2017). Dorsal root ganglion neurons become hyperexcitable and increase expression of voltage-gated T-type calcium channels (Cav3.2) in paclitaxel-induced peripheral neuropathy. Pain.

[8] Sisignano M., Angioni C., Park C.K., Meyer Dos S.S., Jordan H., Kuzikov M. (2016). Targeting CYP2J to reduce paclitaxel-induced peripheral neuropathic pain. Proc Natl Acad Sci U S A.

[9] Zhang H., Dougherty P.M. (2014). Enhanced excitability of primary sensory neurons and altered gene expression of neuronal ion channels in dorsal root ganglion in paclitaxel-induced peripheral neuropathy. Anesthesiology.

[10] Cai Y.Q., Chen S.R., Pan H.L. (2013). Upregulation of nuclear factor of activated T-cells by nerve injury contributes to development of neuropathic pain. J Pharmacol Exp Ther.

[11] Wang C.H., Zou L.J., Zhang Y.L., Jiao Y.F., Sun J.H. (2010). The excitatory effects of the chemokine CCL2 on DRG somata are greater after an injury of the ganglion than after an injury of the spinal or peripheral nerve. Neurosci Lett.

[12] White F.A., Feldman P., Miller R.J. (2009). Chemokine signaling and the management of neuropathic pain. Mol Interv.

[13] Illias A.M., Gist A.C., Zhang H., Kosturakis A.K., Dougherty P.M. (2018). Chemokine CCL2 and its receptor CCR2 in the dorsal root ganglion contribute to oxaliplatin-induced mechanical hypersensitivity. Pain.

[14] Zhang H., Boyette-Davis J.A., Kosturakis A.K., Li Y., Yoon S.Y., Walters E.T. (2013). Induction of monocyte chemoattractant protein-1 (MCP-1) and its receptor CCR2 in primary sensory neurons contributes to paclitaxel-induced peripheral neuropathy. J Pain.

[15] Jung H., Toth P.T., White F.A., Miller R.J. (2008). Monocyte chemoattractant protein-1 functions as a neuromodulator in dorsal root ganglia neurons. J Neurochem.

[16] Sun J.H., Yang B., Donnelly D.F., Ma C., Lamotte R.H. (2006). MCP-1 enhances excitability of nociceptive neurons in chronically compressed dorsal root ganglia. J Neurophysiol.

[17] Van S.J., Reaux-Le G.A., Pommier B., Mauborgne A., Dansereau M.A., Kitabgi P. (2011). CCL2 released from neuronal synaptic vesicles in the spinal cord is a major mediator of local inflammation and pain after peripheral nerve injury. J Neurosci.

[18] White F.A., Sun J., Waters S.M., Ma C., Ren D., Ripsch M. (2005). Excitatory monocyte chemoattractant protein-1 signaling is up-regulated in sensory neurons after chronic compression of the dorsal root ganglion. Proc Natl Acad Sci U S A.

[19] Belkouch M., Dansereau M.A., Reaux-Le G.A., Van S.J., Beaudet N., Chraibi A. (2011). The chemokine CCL2 increases Nav1.8 sodium channel activity in primary sensory neurons through a gbetagamma-dependent mechanism. J Neurosci.

[20] Kao D.J., Li A.H., Chen J.C., Luo R.S., Chen Y.L., Lu J.C. (2012). CC chemokine ligand 2 upregulates the current density and expression of TRPV1 channels and Nav1.8 sodium channels in dorsal root ganglion neurons. J Neuroinflammation.

[21] Zhao R., Pei G.X., Cong R., Zhang H., Zang C.W., Tian T. (2014). PKC-NF-kappaB are involved in CCL2-induced Nav1.8 expression and channel function in dorsal root ganglion neurons. Biosci Rep.

[22] Abbadie C., Lindia J.A., Cumiskey A.M., Peterson L.B., Mudgett J.S., Bayne E.K. (2003). Impaired neuropathic pain responses in mice lacking the chemokine receptor CCR2. Proc Natl Acad Sci U S A.

[23] Gao Y.J., Zhang L., Samad O.A., Suter M.R., Yasuhiko K., Xu Z.Z. (2009). JNK-induced MCP-1 production in spinal cord astrocytes contributes to central sensitization and neuropathic pain. J Neurosci.

[24] Imai S., Ikegami D., Yamashita A., Shimizu T., Narita M., Niikura K. (2013). Epigenetic transcriptional activation of monocyte chemotactic protein 3 contributes to long-lasting neuropathic pain. Brain.

[25] Van Steenwinckel J., Reaux-Le Goazigo A., Pommier B., Mauborgne A., Dansereau M.A., Kitabgi P. (2011). CCL2 released from neuronal synaptic vesicles in the spinal cord is a major mediator of local inflammation and pain after peripheral nerve injury. J Neurosci.

[26] Al-Mazidi S., Alotaibi M., Nedjadi T., Chaudhary A., Alzoghaibi M., Djouhri L. (2018). Blocking of cytokines signalling attenuates evoked and spontaneous neuropathic pain behaviours in the paclitaxel rat model of chemotherapy-induced neuropathy. Eur J Pain.

[27] Pascual M., Vicente M., Monferrer L., Artero R. (2006). The Muscleblind family of proteins: an emerging class of regulators of developmentally programmed alternative splicing. Differentiation.

[28] Kanadia R.N., Urbinati C.R., Crusselle V.J., Luo D., Lee Y.J., Harrison J.K. (2003). Developmental expression of mouse muscleblind genes Mbnl1, Mbnl2 and Mbnl3. Gene Expr Patterns.

[29] Holt I., Jacquemin V., Fardaei M., Sewry C.A., Butler-Browne G.S., Furling D. (2009). Muscleblind-like proteins: similarities and differences in normal and myotonic dystrophy muscle. Am J Pathol.

[30] Wang E.T., Cody N.A., Jog S., Biancolella M., Wang T.T., Treacy D.J. (2012). Transcriptome-wide regulation of pre-mRNA splicing and mRNA localization by muscleblind proteins. Cell.

[31] Blech-Hermoni Y., Ladd A.N. (2013). RNA binding proteins in the regulation of heart development. Int J Biochem Cell Biol.

[32] Konieczny P., Stepniak-Konieczna E., Sobczak K. (2014). MBNL proteins and their target RNAs, interaction and splicing regulation. Nucleic Acids Res.

[33] Hao M., Akrami K., Wei K., De Diego C., Che N., Ku J.H. (2008). Muscleblind-like 2 (Mbnl2) -deficient mice as a model for myotonic dystrophy. Dev Dyn.

[34] Kanadia R.N., Johnstone K.A., Mankodi A., Lungu C., Thornton C.A., Esson D. (2003). A muscleblind knockout model for myotonic dystrophy. Science.

[35] Jia S., Wei G., Bono J., Pan Z., Zheng B., Wang B. (2022). TET1 overexpression attenuates paclitaxel-induced neuropathic pain through rescuing K2p1.1 expression in primary sensory neurons of male rats. Life Sci.

[36] Mao Q., Wu S., Gu X., Du S., Mo K., Sun L. (2019). DNMT3a-triggered downregulation of K2p 1.1 gene in primary sensory neurons contributes to paclitaxel-induced neuropathic pain. Int J Cancer.

[37] Wei W., Liu W., Du S., Govindarajalu G., Irungu A., Bekker A. (2021). A compound mitigates cancer pain and chemotherapy-induced neuropathic pain by dually targeting nNOS-PSD-95 interaction and GABAA receptor. Neurotherapeutics.

[38] Wen C.H., Berkman T., Li X., Du S., Govindarajalu G., Zhang H. (2023). Effect of intrathecal NIS-lncRNA antisense oligonucleotides on neuropathic pain caused by nerve trauma, chemotherapy, or diabetes mellitus. Br J Anaesth.

[39] Yang Y., Wen J., Zheng B., Wu S., Mao Q., Liang L. (2021). CREB participates in paclitaxel-induced neuropathic pain genesis through transcriptional activation of Dnmt3a in primary sensory neurons. Neurotherapeutics.

[40] Du S., Wu S., Feng X., Wang B., Xia S., Liang L. (2022). A nerve injury-specific long noncoding RNA promotes neuropathic pain by increasing Ccl2 expression. J Clin Investig.

[41] Pan Z., Du S., Wang K., Guo X., Mao Q., Feng X. (2021). Downregulation of a dorsal root ganglion-specifically enriched long noncoding RNA is required for neuropathic pain by negatively regulating RALY-triggered Ehmt2 expression. Adv Sci (Weinh).

[42] Wang B., Ma L., Guo X., Du S., Feng X., Liang Y. (2023). A sensory neuron-specific long non-coding RNA reduces neuropathic pain by rescuing KCNN1 expression. Brain.

[43] Zhao J.Y., Liang L., Gu X., Li Z., Wu S., Sun L. (2017). DNA methyltransferase DNMT3a contributes to neuropathic pain by repressing Kcna2 in primary afferent neurons. Nat Commun.

[44] Zhao X., Tang Z., Zhang H., Atianjoh F.E., Zhao J.Y., Liang L. (2013). A long noncoding RNA contributes to neuropathic pain by silencing Kcna2 in primary afferent neurons. Nat Neurosci.

[45] Zhang Y., Wang B., Feng X., Wang H., Gao J., Li X. (2024). RNA-binding protein SYNCRIP contributes to neuropathic pain through stabilising CCR2 expression in primary sensory neurones. Br J Anaesth.

[46] Huang L., Sharma D., Feng X., Pan Z., Wu S., Munoz D. (2024). RALY participates in nerve trauma-induced nociceptive hypersensitivity through triggering Eif4g2 gene expression in primary sensory neurons. Br J Pharmacol.

[47] Han G., Li X., Wen C.H., Wu S., He L., Tan C. (2023). FUS contributes to nerve injury-induced nociceptive hypersensitivity by activating NF-kappaB pathway in primary sensory neurons. J Neurosci.

[48] Xu Y., Gu Y., Wu P., Li G.W., Huang L.Y. (2003). Efficiencies of transgene expression in nociceptive neurons through different routes of delivery of adeno-associated viral vectors. Hum Gene Ther.

[49] Xu Y., Gu Y., Xu G.Y., Wu P., Li G.W., Huang L.Y. (2003). Adeno-associated viral transfer of opioid receptor gene to primary sensory neurons: a strategy to increase opioid antinociception. Proc Natl Acad Sci U S A.

[50] Masuda A., Andersen H.S., Doktor T.K., Okamoto T., Ito M., Andresen B.S. (2012). CUGBP1 and MBNL1 preferentially bind to 3' UTRs and facilitate mRNA decay. Sci Rep.

[51] Batra R., Charizanis K., Manchanda M., Mohan A., Li M., Finn D.J. (2014). Loss of MBNL leads to disruption of developmentally regulated alternative polyadenylation in RNA-mediated disease. Mol Cell.

[52] Zhou H., Xu J., Pan L. (2025). Functions of the Muscleblind-like protein family and their role in disease. Cell Commun Signal.

[53] Byrne B.J., Flanigan K.M., Matesanz S.E., Finkel R.S., Waldrop M.A., D'Ambrosio E.S. (2025). Current clinical applications of AAV-mediated gene therapy. Mol Ther.

[54] Park K.S., Cho Y.I., Mitragotri S., Zhao Z. (2026). Viral vector-based gene therapies in the clinic: an update. Bioeng Transl Med.

[55] Slyk Z., Stachowiak N., Malecki M. (2024). Gene therapy in the light of lifestyle diseases: budesonide, Acetaminophen and Simvastatin modulates rAAV transduction efficiency. Pharmaceuticals.

[56] Wang J.H., Gessler D.J., Zhan W., Gallagher T.L., Gao G. (2024). Adeno-associated virus as a delivery vector for gene therapy of human diseases. Signal Transduct Target Ther.

