# Supplementary material for: RNA-binding protein MBNL2 mitigates neuropathic pain after chemotherapy through destabilizing CCR2 expression in primary sensory neurons
Source: Neurotherapeutics. 2026 Apr 11;23(3):e00905. doi: 10.1016/j.neurot.2026.e00905 (PMC13092599; doi:10.1016/j.neurot.2026.e00905)
Supplement: Multimedia component 1 [file mmc1.docx]

**Supplementary Table 1**. Primers and probes

RT-PCR

Names

Sequences

5′- TCCATCCTGTGCCCACTTTTC-3′

*Mbnl2 F*

5′- TCCCTGCATACCTCCAGTTT-3′

*Mbnl2 R*

*Tuba1a F*

5′-GTGCATCTCCATCCATGTTG-3′

*Tuba1a R*

5′-GTGGGTTCCAGGTCTACGAA-3′

Vector construction

AAV5-MBNL2 F

5′- TGGCCTTGAACGTTGCCC -3′

AAV5- MBNL2 R

5′- CGGTTAGTCTATTAAGACTTTATT -3′

5′GATCCGCAATTTATGTTTCCAGGATTCAAGAGATCCTGGAAACATAAATTGCTTTTTTG-3′

*Mbnl2* shRNA SE

5′AATTCAAAAAAGCAATTTATGTTTCCAGGATCTCTTGAATCCTGGAAACATAAATTGCG -3′

*Mbnl2* shRNA AS

RT: Reverse-transcription; F, Forward; R, Reverse. AS: Antisense; SE: Sense.
