# Supplementary material for: RNA-binding protein MBNL2 mitigates neuropathic pain after chemotherapy through destabilizing CCR2 expression in primary sensory neurons
Source: Neurotherapeutics. 2026 Apr 11;23(3):e00905. doi: 10.1016/j.neurot.2026.e00905 (PMC13092599; doi:10.1016/j.neurot.2026.e00905)
Supplement: Multimedia component 2 [file mmc2.docx]

**Supplementary Table 2**: Locomotor function

| Treatment groups | Placing | Grasping | Righting |
| --- | --- | --- | --- |
| AAV5-GFP + Vehicle (male) | 5 (0) | 5 (0) | 5 (0) |
| AAV5-GFP + PTX (male) | 5 (0) | 5 (0) | 5 (0) |
| AAV5-MBNL2 + Vehicle (male) | 5 (0) | 5 (0) | 5 (0) |
| AAV5-MBNL2+PTX (male) | 5 (0) | 5 (0) | 5 (0) |
| KI + Vehicle (male) | 5 (0) | 5 (0) | 5 (0) |
| KI + PTX (male) | 5 (0) | 5 (0) | 5 (0) |
| cKI + Vehicle (male) | 5 (0) | 5 (0) | 5 (0) |
| cKI + PTX (male) | 5 (0) | 5 (0) | 5 (0) |
| Mbnl2^f/f^ (male) | 5 (0) | 5 (0) | 5 (0) |
| cKD (male) | 5 (0) | 5 (0) | 5 (0) |
| KI + Vehicle (female) | 5 (0) | 5 (0) | 5 (0) |
| KI + PTX(female) | 5 (0) | 5 (0) | 5 (0) |
| cKI + Vehicle (female) | 5 (0) | 5 (0) | 5 (0) |
| cKI + PTX (female) | 5 (0) | 5 (0) | 5 (0) |
| Mbnl2^f/f^ (female) | 5 (0) | 5 (0) | 5 (0) |
| cKD (female) | 5 (0) | 5 (0) | 5 (0) |
|  |  |  |  |

n = 8 mice per group; 5 trials; Mean (SE). GFP: enhanced green fluorescent protein. PTX: paclitaxel. AAV5: recombinant adeno-associated virus type 5. KI: Rosa26*^Mbnl2^* knock-in mice. cKI: sensory neuron-specific inducible conditional MBNL2 knock-in mice. cKD: Sensory neuron-specific inducible conditional MBNL2 knockdown mice.
